# Identification and validation of TNFRSF4 as a high-profile biomarker for prognosis and immunomodulation in endometrial carcinoma

**DOI:** 10.1186/s12885-022-09654-6

**Published:** 2022-05-13

**Authors:** Heng Ma, Peng-hui Feng, Shuang-ni Yu, Zhao-hui Lu, Qi Yu, Jie Chen

**Affiliations:** 1 Department of Pathology, Peking Union Medical College Hospital, Peking Union Medical College and Chinese Academy of Medical Science, Beijing, 100730 China; 2 Department of Obstetrics and Gynecology, Peking Union Medical College Hospital, Peking Union Medical College and Chinese Academy of Medical Science, Beijing, 100730 China

**Keywords:** TNFRSF4_1_, biomarker_2_, prognosis_3_, Tumor microenvironment_4_, Endometrial carcinoma_5_

## Abstract

**Background:**

The interaction between tumor microenvironment (TME) and tumors offers various targets in mounting anti-tumor immunotherapies. However, the prognostic biomarkers in endometrial carcinoma (EC) are still limited. Here, we aimed to analyze the TME features and identify novel prognostic biomarkers for EC.

**Methods:**

ESTIMATE, CIBERSORT, protein-protein interaction (PPI) network, univariate and multivariate Cox regression, and functional enrichment analysis were performed to identify immune- and survival-related hub genes as well as possible molecular mechanisms. The limma package and deconvolution algorithm were adopted to estimate the abundance of tumor-infiltrating immune cells (TICs) and their relationship with the target gene. In the validation section, tissue microarrays (TMAs) of EC and multiplex immunohistochemistry (m-IHC) were evaluated to validate the expression of TNFRSF4, and its correlation with immune markers, including CD4, CD8, and FOXP3. Besides, the receiver operating characteristic (ROC) curve was plotted to determine the diagnostic performance of TNFRSF4, CD4, CD8, and FOXP3 in EC.

**Results:**

Two genes, *TNFRSF4* and *S1PR4*, were screened out from 386 intersection differential expression genes (DEGs) shared by ImmuneScore and StromalScore in EC. Highlighted by *TNFRSF4*, we found that it was not only positively correlated with the TICs (mainly CD4^+^ T cells, CD8^+^ T cells, and Tregs) but significantly related to the prognosis in patients of EC, both verified by data from The Cancer Genome Altas (TCGA)-EC database and clinical samples. At the same time, the expression trend of *TNFRSF4* was further confirmed by an integrated meta-analysis based on six microarrays from the Gene Expression Omnibus database (GEO).

**Conclusions:**

Collectively, *TNFRSF4*, a previously unrecognized key player in EC, could serve as a potential biomarker for prognosis prediction and immunomodulation of EC.

**Supplementary Information:**

The online version contains supplementary material available at 10.1186/s12885-022-09654-6.

## Introduction

Endometrial carcinoma (EC), the second most common carcinoma of the female genital tract globally [1], originates from the inner epithelial lining of the uterus. The most common clinical symptoms of EC include abnormal vaginal bleeding or discharge, abdominal pain, especially for postmenopausal women. According to estimates in 2020, there will be 65,620 new cases of EC diagnosed, 12,590 deaths caused by EC in the United States [2], and both morbidity and mortality rates have exhibited a sustained rise, particularly in cases among women under the age of 50 years (1.3% per year from 2007 ~ 2016) [3]. Although the prognosis of EC is well generally, however, most of the patients with grade III endometrioid endometrial carcinoma (EEC) and non-EEC (include serous clear cell, undifferentiated carcinomas and carcinosarcomas), who are confronted with enormous risks for metastasis or systemic recurrence [4]. Once it happens, the median survival for EC patients is about 4 months. In addition, novel therapies such as optimized individual treatment based on molecular classification are not well defined in terms of surgical staging, surveil-lance scheduling and adjuvant therapy [5, 6].

The tumor microenvironment (TME) is necessary for the living of tumor cells. It, either directly or indirectly, affects tumor occurrence and development through maintaining a quiescent state of immune contexture [7], promoting tumor angiogenesis [8], changing biological features of carcinoma [9], or even regulating the cancer stem cell activity [10, 11]. The orchestration between tumor and TME not only engenders tumor expansion, but also increased tumor mutation probability by creates heterologous TME. Additionally, TME is inextricably linked to tumor immune suppression or activation. In addition to tumor cells, the stromal cells, chemokines, and cytokines, TME comprises innate immune cells (including macrophages, neutrophils, dendritic cells, myeloid-derived suppressor cells, and natural killer cells) and adaptive immune cells (including T cells and B cells) [12]. Both populations of immune cells innately modulate tumor cell-intrinsic and extrinsic processes within TME. To date, some technique has been devoted to characterize the crosstalk between the tumor and its microenvironments, such as multiplex immunohistochemistry (m-IHC) and bulk tissue microarrays (TMAs) [13–15]. ESTIMATE (estimation of stromal and immune cells in malignant tumor tissues using expression data) is a highly rated tool developed by Kosuke Yoshihara and his co-workers for predicting tumor purity mainly based on the ssGSEA algorithm [16], which has helped us to estimate the abundance of tumor-infiltrating lymphocytes (TILs) and subsequently stratify patients for predicting clinical outcomes [17]. Increasing evidence has elucidated that the difference in the efficacy of tumor immunotherapy was mainly due to the heterogeneity of TME [18]. Even though most of these valuable methods and theories have been well applied and tested in many cancers, many works are still needed to make sense of the correlation between TME biological characteristics and the aberrant expression of immune-related genes in EC. Therefore, more reliable and effective surrogate biomarkers are left to be explored to predict the pathological behavior of EC and enable the stratification of patients according to immune-related criteria for improving prognostic and selecting appropriate adjuvant therapy to guide clinical decisions.

Here, we used the ESTIMATE algorithms and the tumor-infiltrating immune cells (TICs) profile to perform a comprehensive analysis of TME and detect related gene expression in patients with EC, through which *TNFRSF4* was identified to be associated with the prognosis of EC as a crucial indicator of TME remodeling. Moreover, to support our findings, clinical specimens were applied to validate the expression of *TNFRSF4* in EC and adjacent normal tissues. The correlations of *TNFRSF4* with clinicopathologic features and immune-related markers (including CD4, CD8, and FOXP3) were evaluated. Overall, our results indicated that *TNFRSF4* might be a biomarker of prognostic values, as a crucial role in TME of EC.

## Methods

The research thoughts and structures of this study were presented in Supplementary Fig. 1.

### Raw data acquisition and processing

Transcriptome RNA-seq data of 587 EC cases (552 tumor samples and 35 controls) and corresponding clinical information were obtained from the TCGA database (https://portal.gdc.cancer.gov/).

### Estimation evaluation and DEGs generation

Estimate algorithm was applied to determine the immune-stromal component in TME of each sample utilizing estimate package in R software (https://r-forge.r-project.org/), the final results of which were calculated as StromalScore, ImmuneScore, and ESTIMATEScore, representing the ratio of stromal cell, immune cell, and the summation of both cells, respectively [19]. Based on the median of the StromalScore and ImmuneScore, samples were divided into either the high or low score groups, and survival analysis was carried out by R software loaded with survival and survminer packages. Five hundred forty one tumor samples were finally analyzed with complete survival information, and the survival curve was produced by the Kaplan-Meier method and compared between groups by the log-rank test. At the same time, clinical data were acquired and analyzed to ascertain the correlation between scores and clinical parameters. Then, differentially expressed genes (DEGs) of StromalScore and ImmuneScore between the high and low score groups were screened out by limma package [20] with false discovery rate (FDR) < 0.05 and |log fold change (FC)| > 1.

### Functional enrichment analysis

DEGs were displayed and processed as heatmaps according to the expression level using the pheatmap package for clustering (https://CRAN.R-project.org/package=pheatmap). To determine the functional enrichment for gene ontology (GO) and Kyoto Encyclopedia of Genes and Genomes (KEGG) pathways analysis, R packages (clusterProfiler, enrichplot, org.Hs.eg.db, and ggplot2) were utilized to identify gene functions and achieve visualization for functional profiles, where q-value of less than 0.05 were considered significantly enriched.

### PPI network construction and cox regression analysis

Protein-protein interaction (PPI) network was produced through the online analytical database STRING (https://string-db.org/cgi/input.pl) to clarify the interactive relation among genes (only were the edges and nodes kept when the confidence of interactive relationships > 0.4). To further confirm the highly interconnected genes, the MCODE plugin of Cytoscape (https://cytoscape.org/) was applied, and the interactive hub cluster was selected with 56 hub genes and 794 edges (degree cutoff = 0.2, K-core = 2). Additionally, Cox regression analysis was conducted by using R package survival. Genes with *P*-value < 0.05 were displayed.

### Gene set enrichment analysis

H.all.v7.2.symbols Hallmark and C7.all.v7.2.symbols immunological signatures gene sets were downloaded from the Molecular Signatures Database (http://software.broadinstitute.org/gsea/msigdb/index.jsp). All gene sets were analyzed utilizing the software of GSEAS-4.0 downloaded from Broad Institute.

### TICs profile

The limma package in R was applied to normalize the data to evaluate the proportion of TICs, and then a standardized gene expression profile was uploaded to CIBERSORT. The deconvolution algorithm was adopted to estimate the TIC abundance [21]. Only 235 tumor samples with *P*-value < 0.05 were screened out by quality filtering and applied to the following analysis. Meanwhile, single sample geneset enrichment analysis (ssGSEA) was also introduced here to investigate the immune infiltration landscape of T cell subsets of EC to determine their association with the expression of *TNFRSF4*.

### Receiver operating characteristic (ROC) curves

To determine the diagnostic values of interesting genes in EC, pROC package [22] was used to plot ROC curves, and the values of areas under the curves (AUCs) and corresponding 95% CI were calculated in R.

### A meta-analysis based on GEO microarrays to determine the expression of *TNFRSF4*

The GEO database was searched to retrieve the microarray data on the expression of *TNFRSF4* in EC with the retrieval strategies as follows ((‘endometrial’ OR ‘endometrium’) AND (‘cancer’ OR ‘cancerous’ OR ‘carcinoma’ OR ‘carcinomatous’ OR ‘neoplasm’ OR ‘malignancy’ OR ‘tumor’ OR ‘tumors’) AND (‘mRNA’ OR ‘RNA’ OR ‘coding RNA’). Additionally, the inclusion criteria were set to 1) patients diagnosed as EC were involved, 2) the experiments were composed of tumor samples and normal controls, and 3) the expression profiling data of *TNFRSF4* was available. The expression profiling data of *TNFRSF4* obtained from the queried microarrays were integrated for comparisons between the two groups. Subsequently, a comprehensive meta-analysis by the meta-package was adopted to evaluate the data from all selected microarrays, for which the forest plot presented the expression of *TNFRSF4*. Furthermore, the DerSimonian-Laird estimator for τ^2^ and I^2^ parameters were utilized to determine whether to adopt the corresponding model (random-effects or fixed-effects models) for the pooled estimate. The sensitivity analysis was performed to evaluate the heterogeneity among all investigated studies. Begg’s test and funnel plot were carried out to assess the potential publication bias.

### Tissue microarray and ethical statement

TMAs were purchased from Shanghai Outdo Biotech Co. Ltd. (panel HUteA020CS01, HUteA045PG01, and HUteA060CS01). These TMAs, composed of 121 cores (85 carcinomas and 36 adjacent tissue), the detailed information was displayed in Supplementary Table 1. The research scheme of this study has been reviewed and approved by the Ethics Committee of Peking Union Medical College Hospital (ethics, S-K973).

### Immunohistochemistry (IHC) and evaluation of immunostaining

IHC staining was performed as described previously [23, 24]. Briefly, TMAs specimens were deparaffinized, hydrated, and subjected to heat-mediated antigen retrieval with EDTA buffer at 95 °C for 10 min. After endogenous peroxidase was quenched and tissue blocked, TMAs sections were incubated with primary antibodies (listed in Supplementary Table 2) and coated at 4 °C overnight, then with biotin-conjugated secondary reagents for 30 min. At the end of the staining, whole TMAs slides were digitally scanned at × 400 using a NanoZoomer S360 (Hamamatsu, Japan) for visualization. Two experienced pathologists independently determined the semi-quantitative immunoreactive scores to evaluate the immunostaining. Given that TNFRSF4, CD8, CD4, and FOXP3 were expressed in TILs, its scoring has used the method of Erminia Massarelli [25] by counting positive cells in the five random square areas at 400× magnification, and the expression of each marker was recorded as the density of positive cells/mm^2^.

### Multiplex immunohistochemistry (m-IHC) and immunofluorescence

M-IHC staining was performed on TMAs serial sections according to the manufacturer’s protocol (Opal Multiplex IHC Assay Kit; Akoya Biosciences, MA). Briefly, TMAs specimens were baked at 65 °C for 1 h, dewaxed with xylene, rehydrated with a graded series of ethanol solutions ethanol. For epitope retrieval, slides were placed in a microwave with AR6 antigen retrieval buffer for 45 s at 100% power and an additional 15 min at 20% power. After blocking, slides were incubated with the first primary antibody for 1 h at room temperature, followed by Polymer HRP Ms. + Rb and opal fluorophore working solution for 10 min at room temperature, respectively. Slides were stripped with microwave treatment before repeating until all targets of interest were detected. Apply DAPI working solution for 5 min at room temperature and covered with mounting medium, then representative images (Supplementary Fig. 2) from each sample were acquired and analyzed by the Vectra Polaris multispectral slide scanner and supporting software (Akoya Biosciences).

### Statistical analysis

SPSS (Version. 22, Chicago, IL, USA) was used for statistical analyses. Shapiro-Wilk test, combined with normality plots, was applied to determine the normal distribution. For unpaired samples, comparisons between two groups were analyzed by the Mann-Whitney test or Student’s t-test. As for paired samples, the Wilcoxon rank-sum test was applied. Besides, multiple comparisons were carried out based on the Kruskal Wallis rank-sum test to compare groups’ differences. The two-sided Person chi-squared test or Spearman rank correlation test was used to evaluate the relationship between TNFRSF4 IHC scores and immune-related gene expression levels. Disease-specific survival (DSS) was used to analyze the prognostic impact of TNRSF4, which is defined as the time between the date of surgery and the date of death caused by EC. The Kaplan-Meier analysis by the log-rank test. To estimate the prognostic impact of individual continuous gene expression variables and independent prognostic factors, univariate and multivariate Cox regression analyses were performed, respectively. Statistical significance was defined when *P*-value was less than 0.05.

## Results

### Clinicopathologic significance of the estimate scores

**Fig. 1:**
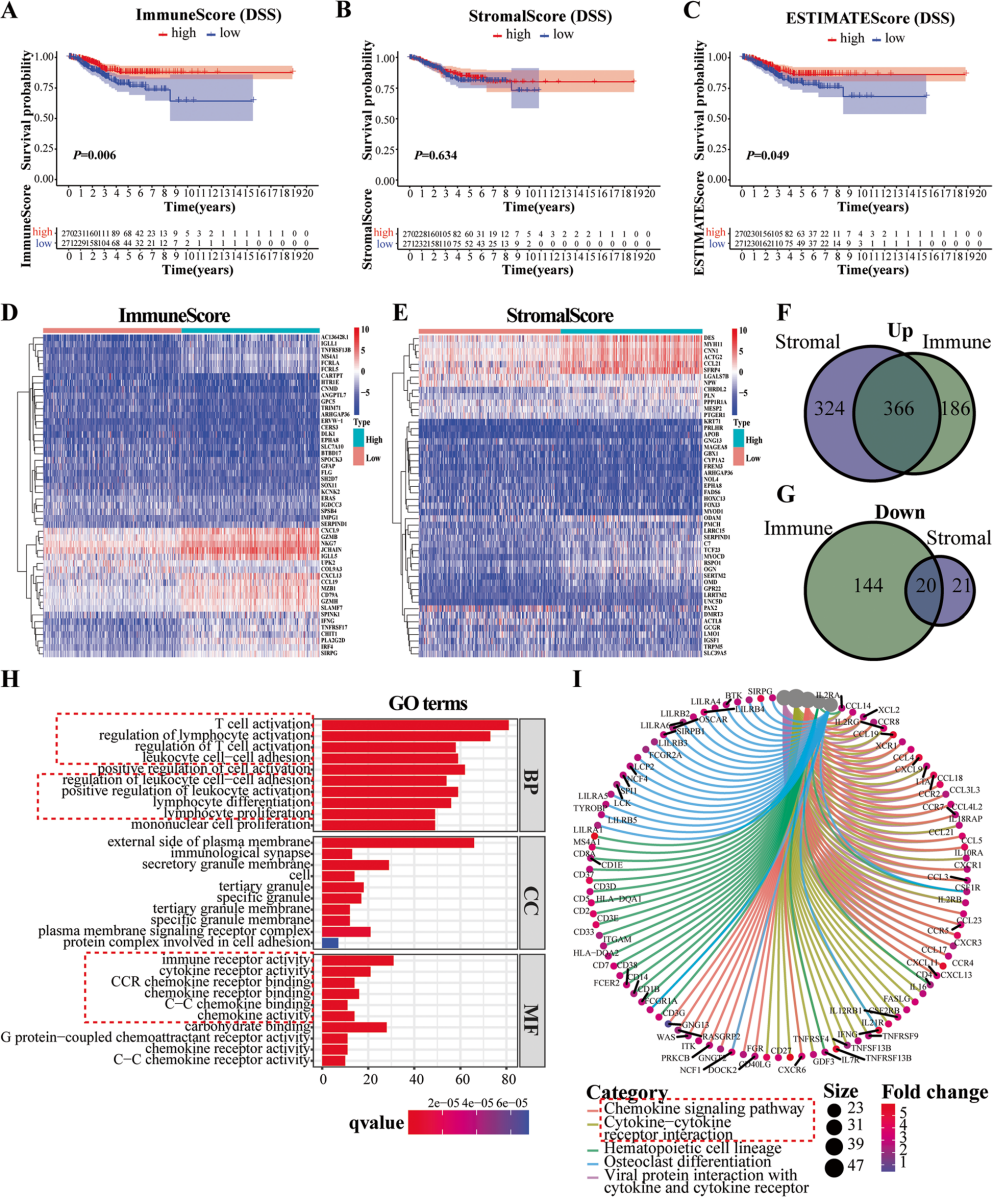
Disease-specific survival (DSS) probability correlated with Estimate scores, and screening of differentially expressed genes as well as functional enrichment. **A** Comparison of the DSS rate between the high or low ImmuneScore groups in EC (*P* = 0.029, *n* = 541). **B** DSS for EC patients with high or low StromalScore (*P* = 0.436, *n* = 541). **C** Comparison of the DSS rate between the high or low ESTIMATEScore groups in EC by log-rank test (*P* = 0.361, *n* = 541). **D** The top 50 differentially expressed genes of EC grouped by ImmuneScore, displayed by heatmap. Rownames represented the gene names, the clustering characteristics of which were analyzed according to the expression patterns of DEGs. **E** Heatmap of the top 50 differentially expressed genes generated by comparison between high or low StromalScore groups, with FDR < 0.05 and |log FC| > 1. The color stood for the expression level of DEGs. **F**, **G** Genes shared by ImmuneScore and StromalScore, displayed by Venn plots (Upper panel for the up-regulated genes and lower panel for the down-regulated ones). **H** Bar plots for the top 10 enriched terms of biological process, cellular component, and molecular function of 386 DEGs in GO enrichment, respectively, where q value < 0.05 were regarded to be enriched significantly. **I** Circos plot for functional classification of KEGG with the identified categories displayed, the KEGG pathway database is granted by Kanehisa laboratories [26]

To reveal the relationship between the component of immune or stromal cells and the clinical outcomes, survival analysis was performed for ImmuneScore, StromalScore, and ESTIMATEScore, respectively. As presented in Fig. 1A, C, the percentage of immune components and ESTIMATEScore were positively correlated with the disease-specific survival rate despite that StromalScore made no difference (Fig. 1B). These results manifested that the immune composition in TME was a more reliable indicator of the prognosis for EC patients. We further estimate the correlation between the three scores and clinicopathologic parameters. The results demonstrated that tumors of EC patients over 60 years old had lower StromalScore than younger patients (*P* < 0.05) even though ImmuneScore and ESTIMATEScore did not differ in terms of age as shown in Supplementary Fig. 3A, B, C. As for the pathological grade, stage and histology, it was worth noting that our data didn’t exhibited consistently decreasing trends in the three scores as tumor progressed, and not all comparisons among groups with different clinicopathological variables were statistically significant (Supplementary Fig. 3D, E, F, G, H, I, J, K, M). These results implied that advanced age (> 60) and higher grade of EC, mainly grade III, tended to have less immune and stromal component, possibly signified poor prognosis.

### DEGs shared by immunescore and stromalscore and functional enrichment

In order to explore the variation of gene profile in the wake of the alteration of the immune or stromal component in TME, we compared the expression level of genes between the high- and low-score groups. Among them, 716 DEGs (552 up-regulated and 164 down-regulated) were determined regarding ImmuneScore, 731 DEGs were screened out from StromalScore, which mainly consisted of highly expressed genes. As shown in the heatmaps, the top 50 DEGs were displayed based on ImmuneScore and StromalScore, and the gene expression pattern showed an apparent difference between groups (Fig. 1D, E). After the intersection, 386 genes were shared in common by ImmuneScore and StromalScore, with 366 up-regulated and the rest down-regulated (Fig. 1F, G). Then, functional enrichment analysis was carried out based on these overlapped genes. Our results indicated that the identified genes were chiefly enriched in the regulation of lymphocyte activation, differentiation, and proliferation in the biological process (BP). About the cellular component (CC) of GO analysis, these genes encoding proteins were the main components of the external side of the plasma membrane, immunological synapse, granule membrane, and protein complex involved in cell adhesion. As for molecular function (MF), the DEGs prevailingly enrolled in immune or cytokine receptor activity (Fig. 1H). KEGG analysis revealed that the chemokine signaling pathway and cytokine-cytokine receptor interaction were most relevant to these DEGs (Fig. 1I). It thus appeared that the overall function of these DEGs primarily focused on immune-related activities, which essentially suggested that the involvement of immune modulation was a notable feature of TME in EC.

### Intersection analysis of PPI network and cox regression

**Fig. 2:**
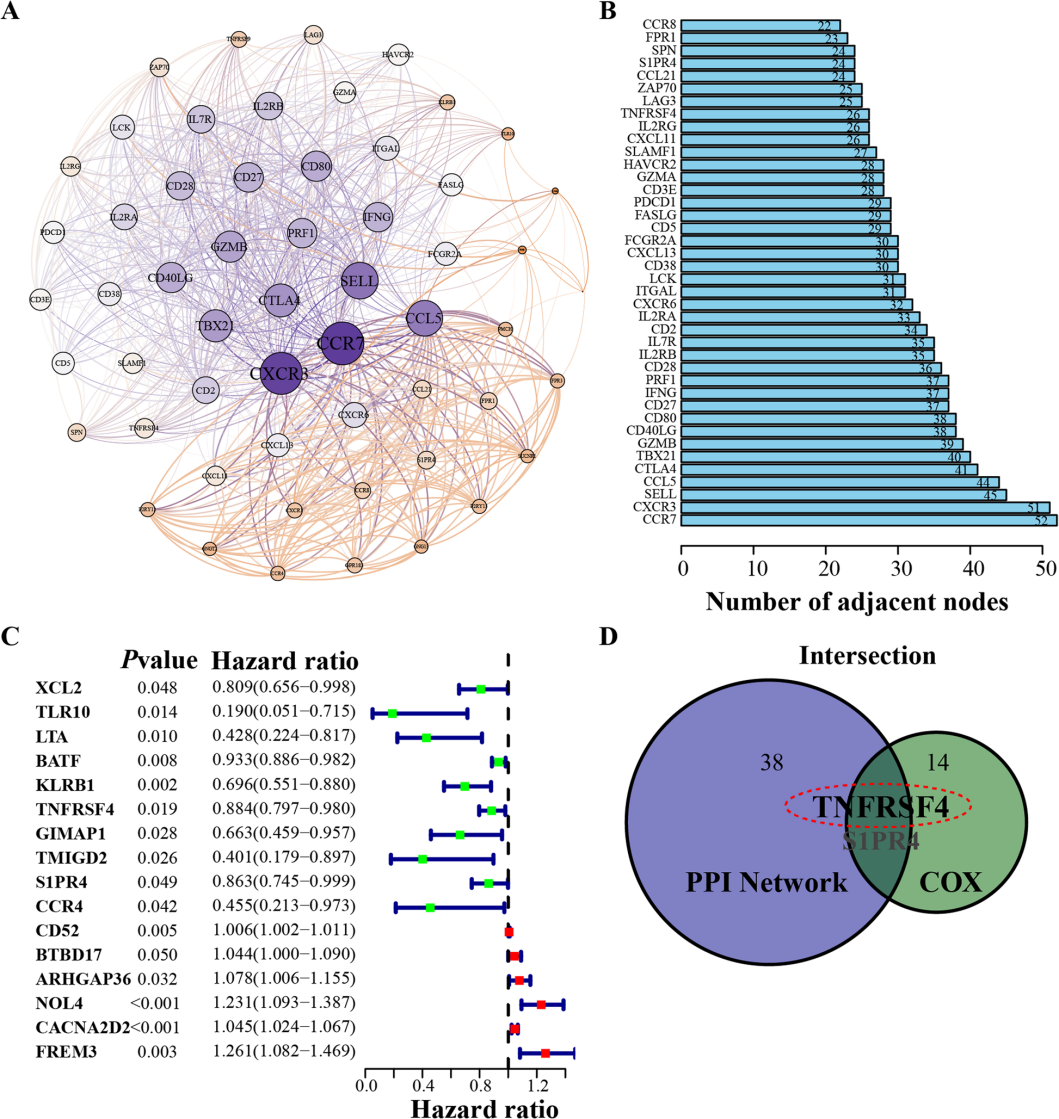
PPI network and univariate Cox regression analysis to determine the target gene. **A** The interactive network of hub clusters, filtered by MCODE analysis. Each node represented a gene. Edges among genes symbolized the interconnected relationships among genes. 56 genes were involved in the cluster. **B** The number of nodes ordered the top 40 genes. **C** Univariate Cox regression analysis with 386 DEGs, correlated with survival probability in EC with *p* < 0.05. **D** Overlapped genes, shared by the top 40 genes in PPI and 16 prognosis-related genes

To elucidate the underlying mechanism, we explored the PPI network constructed by the STRING database, and thus, the interaction between genes was identified. On this basis, the MCODE plugin of Cytoscape software was applied to seek the hub gene cluster. Finally, 56 genes were identified with 794 edges, displayed in Fig. 2A**,** and the bar plot in Fig. 2B showed the top 40 genes, ranked by the number of adjacent nodes. Additionally, to evaluate the prognostic impact of 386 DEGs in EC, univariate Cox regression analysis was performed. In order to present the survival-related genes visually, we are considered introducing the so-called forest plot and the results manifested that the expression level of 16 genes was highly correlated with the survival outcome (Fig. 2C). Subsequently, two genes, *TNFRSF4* and *S1PR4*, were screened out after intersection analysis based on the above PPI network and univariate Cox regression analysis (Fig. 2D).

### Prognostic significance and indicative role in TME remodeling of *TNFRSF4* in EC

**Fig. 3:**
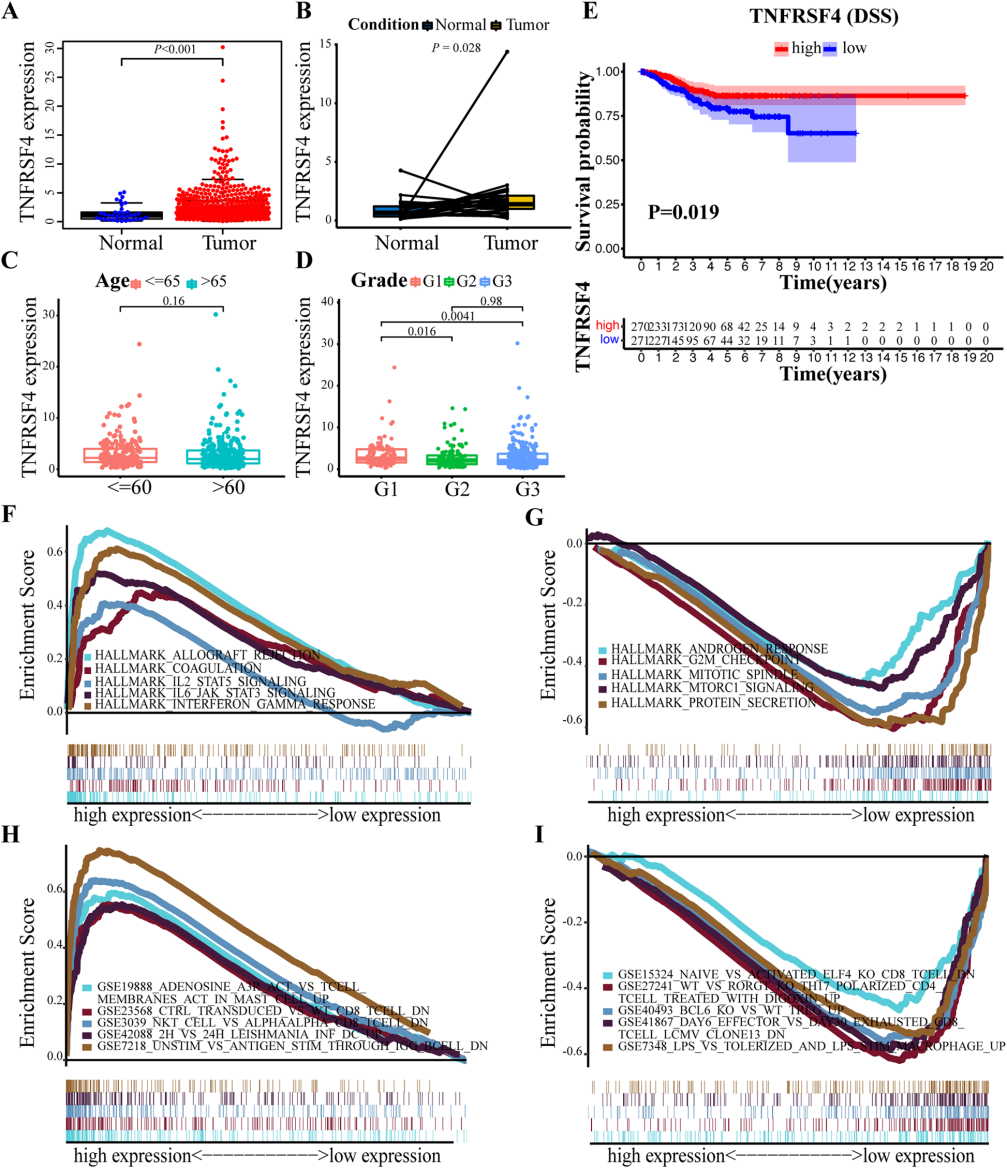
The expression level and GSEA analysis of *TNFRSF4* and its clinicopathologic significance. **A** Expression level of *TNFRSF4* in the normal and tumor tissues (*P* < 0.001). **B** Differential analysis for paired samples to detect the expression of *TNFRSF4*. Lines connected the dots on behalf of pairs of samples from either normal or tumor tissues from a common patient. **C** Differential expression of *TNFRSF4* of samples, grouped by age. **D** The correlation of the expression of *TNFRSF4* compared among groups classified by pathological grade using the Kruskal Wallis rank-sum test. **E** Survival probability analysis by DSS for EC patients with different *TNFRSF4* expression. Patients were recognized as high or low expression according to the comparison with the median expression level. *p* = 0.026. **F** The involved gene sets in HALLMARK collection by the high *TNFRSF4* expression sample. The line chart of the upper part represented the enrichment score of the gene sets. Each line described one particular gene set, distinguished by a unique color, and five leading gene sets were displayed in the plot. **G** The enriched gene sets in HALLMARK by samples with low *TNFRSF4* expression. **H** Enriched gene sets in immunologic signatures by samples of high *TNFRSF4* expression. **I** Enriched gene sets in immunologic signatures by the low *TNFRSF4* expression

**Fig. 4:**
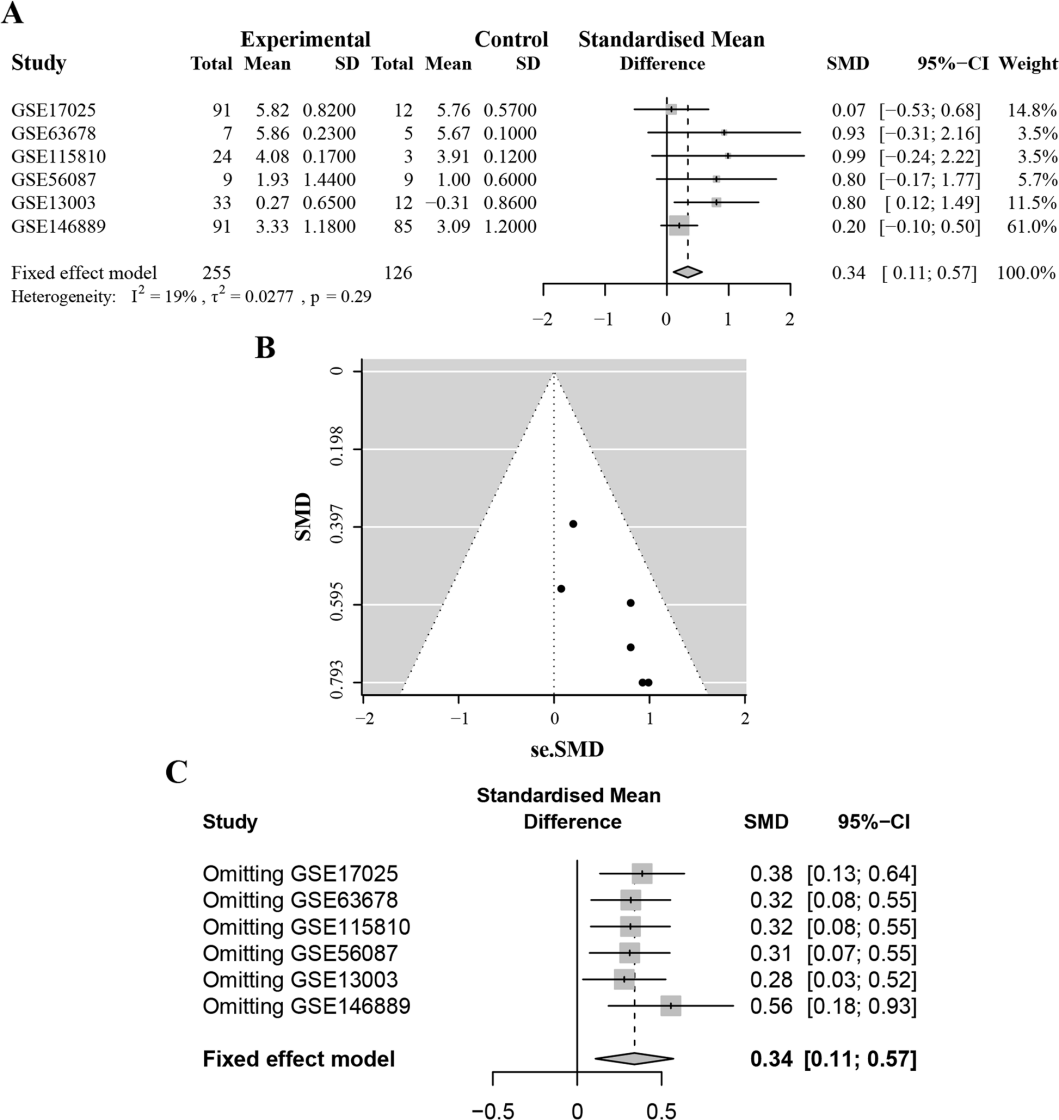
A meta-analysis of the expression of *TNFRSF4* from six collected microarrays. **A** The forest plot for the pooled SMD of 0.34 (95% CI: 0.11, 0.57) without a degree of heterogeneity (I^2^ = 19%, τ^2^ = 0.0277, *p* = 0.29). **B** The funnel plot to assess the possible publication bias of the GEO datasets by Begg’s test with *p* = 0.348. **C** The sensitivity analysis for the GEO microarrays results

Since *S1PR4* is not differentially expressed between paired/unpaired ECs and adjacent normal tissues (Supplementary Fig. 4A, B), *TNFRSF4* was selected for follow-up studies. *TNFRSF4*, barely reported on EC but was a high-profile target on other cancers. *TNFRSF4* was first discovered on the surface of activated CD4^+^ T cells, which played a vital role in immune regulation in multiple cancers as a crucial immune checkpoint. In the following analysis, our data indicated that *TNFRSF4* showed higher expression levels in tumor tissues than normal tissues either in unpaired or paired samples (*P* < 0.001 and *P* = 0.028, respectively), as shown in Fig. 3A, B. To confirm the expression of this gene, six microarrays were eventually involved in the current study from the GEO database, as displayed in Supplementary Table 3. In total, 255 tumor samples and 126 normal tissues were included. Regarding the expression trends of *TNFRSF4*, a meta-analysis was carried out to quantify its expression according to these datasets accurately. The calculation results were presented in Fig. 4A, for which the fixed-effects model was applied given the existed homogeneity (I^2^ = 19%, τ^2^ = 0.0277, *P* = 0.29). Results also indicated that the expression of *TNFRSF4* was dramatically augmented in the EC group (SMD = 0.34, 95% CI [0.11; 0.57], Z = 2.87, *P* = 0.0041). The publication bias was also assessed, as displayed in Fig. 4B. The funnel plot indicated no bias of the publications (z = 0.94, *P* = 0.348). Later, a sensitivity test was generated, and no items were determined to exert possible effects on the results (Fig. 4C). Taken together, it was reasonable to determine the up-regulated expression trend of *TNFRSF4* in EC tissues.

Interestingly, the expression level decreased as aged when compared with the younger group (Fig. 3C). Similarly, tumors of grade II or III exhibited a consistently less amount of *TNFRSF4* when compared with tumors of grade I (Fig. 3D). More importantly, high expression of *TNFRSF4* was significantly associated with a better prognosis in EC based on DSS analysis (*P* = 0.019) (Fig. 3E).

Furthermore, on multivariate analysis, the LASSO regression model was used to reduce the potential collinearity problem and 4 variables were identified (i.e. TNFRSF4, grade, stage, histology), the optimal tuning parameter (λ, lambda.min = 0.015) was selected by cross-validation (Supplementary Fig. 5A,B,). The results suggested that there is no multi-collinearity, and the multivariable Cox regression analysis showed that positive expression of TNFRSF4 was a prognostic factor in terms of improved DSS (HR = 0.317, 95% CI 0.114–0.878, *P* = 0.027) independent of stage, grade, histology (Supplementary Table 4). Additionally, we also analyzed the distribution among the EC molecular subtype (including POLE, microsatellite-instable/hypermutated (MSI), Copy-Number Low and Copy-Number High) based on TCGA categories. Results show that MSI tumors exhibited higher relative frequencies of positive TNFRSF4 expression than did CN-low tumors. The distribution of TNFRSF4 expression among the four EC molecular subgroups from low to high is POLE, CN-low, CN-high and MSI (Supplementary Fig. 4C).

Then, GSEA was implemented for the high and low-expression groups of *TNFRSF4*. As shown in Fig. 3F, the genes in the *TNFRSF4* high-expression group were chiefly enriched in immune-related activities, such as *IFN-γ* response, *IL-2/STAT5* signaling, and *IL-6/JAK/STAT3* signaling. In terms of the *TNFRSF4* low-expression group, the genes were mainly involved in the G2M checkpoint, mitotic spindle, *mTORC1* signaling, and protein secretion (Fig. 3G). For the C7 collection defined by MSigDB, the immunologic gene sets and multiple immune functional gene sets were enrolled in the high and low *TNFRSF4* expression groups (Fig. 3H, I). These results suggested that *TNFRSF4* might be a potential indicator for the status of TME.

### Correlation of *TNFRSF4* with the abundance of TICs

**Fig. 5:**
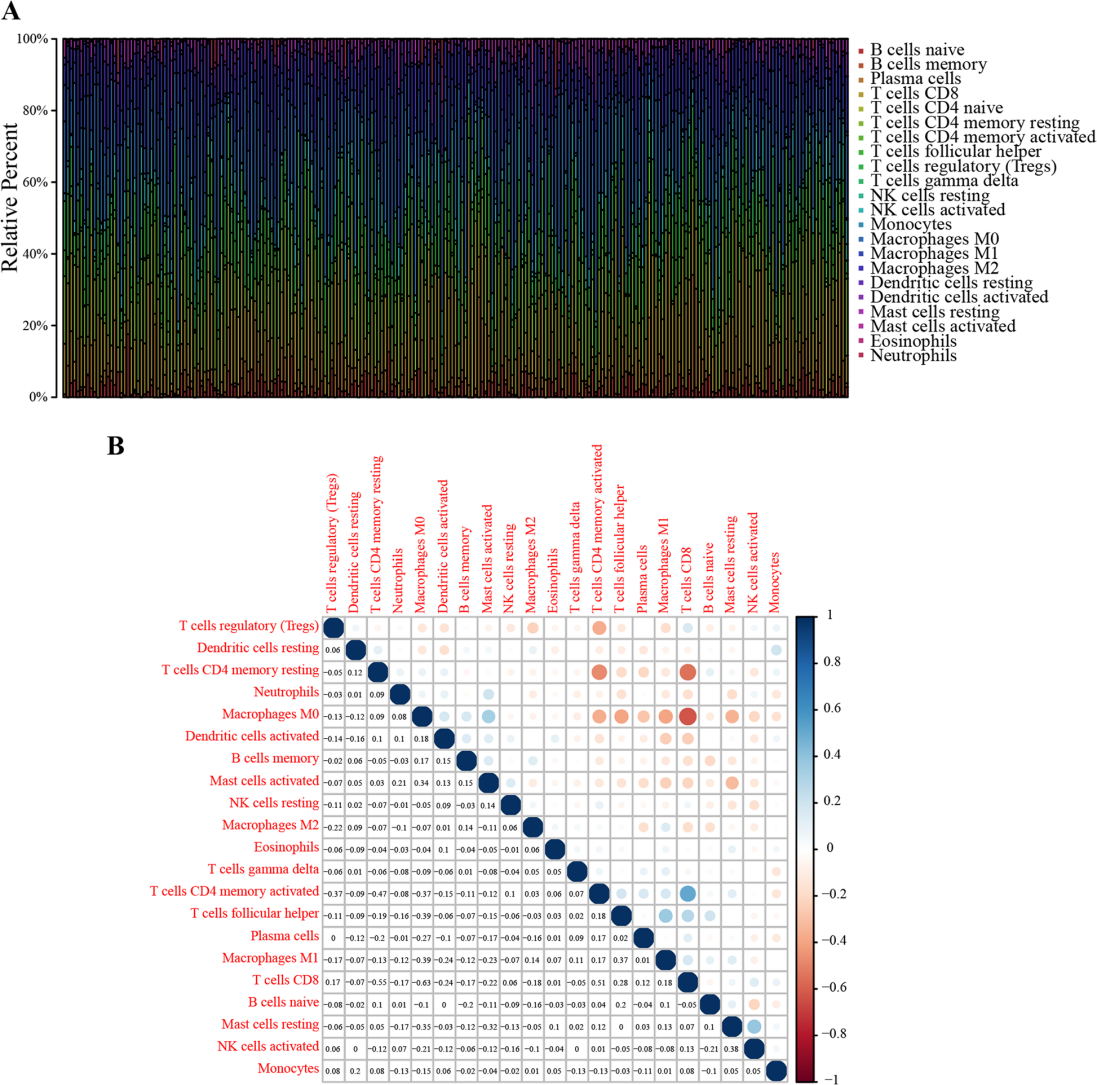
TICs profile in EC samples and correlation analysis. **A** Bar plot described the component of 22 types of TICs in EC tumor samples. Each color represented a kind of immune cell. **B** Heatmap showed the correlation among TICs and color and sizes of each dot indicated correlation coefficient between two kinds of cells

Given the above findings, in order to detect the pertinence relation of TNFRSF4 expression with the immune microenvironment, the component of TICs of each sample was further estimated using the CIBERSORT algorithm. 22 kinds of immune cell profiles in EC samples were identified, as shown in the bar plot (Fig. 5A). It was observed that immune cells in EC were mainly composed of T cells and macrophagocytes. Besides, the correlation among the immune cells was also displayed. The results showed that the proportion of CD8^+^ T cells was negatively correlated with the presence of CD4^+^ memory resting T cells and M0 macrophagocytes (correlation coefficient = − 0.55 or − 0.63, respectively). Conversely, the content of CD8^+^ T cells was positively related to the scale of CD4^+^ memory-activated T cells, as seen in Fig. 5B.

**Fig. 6:**
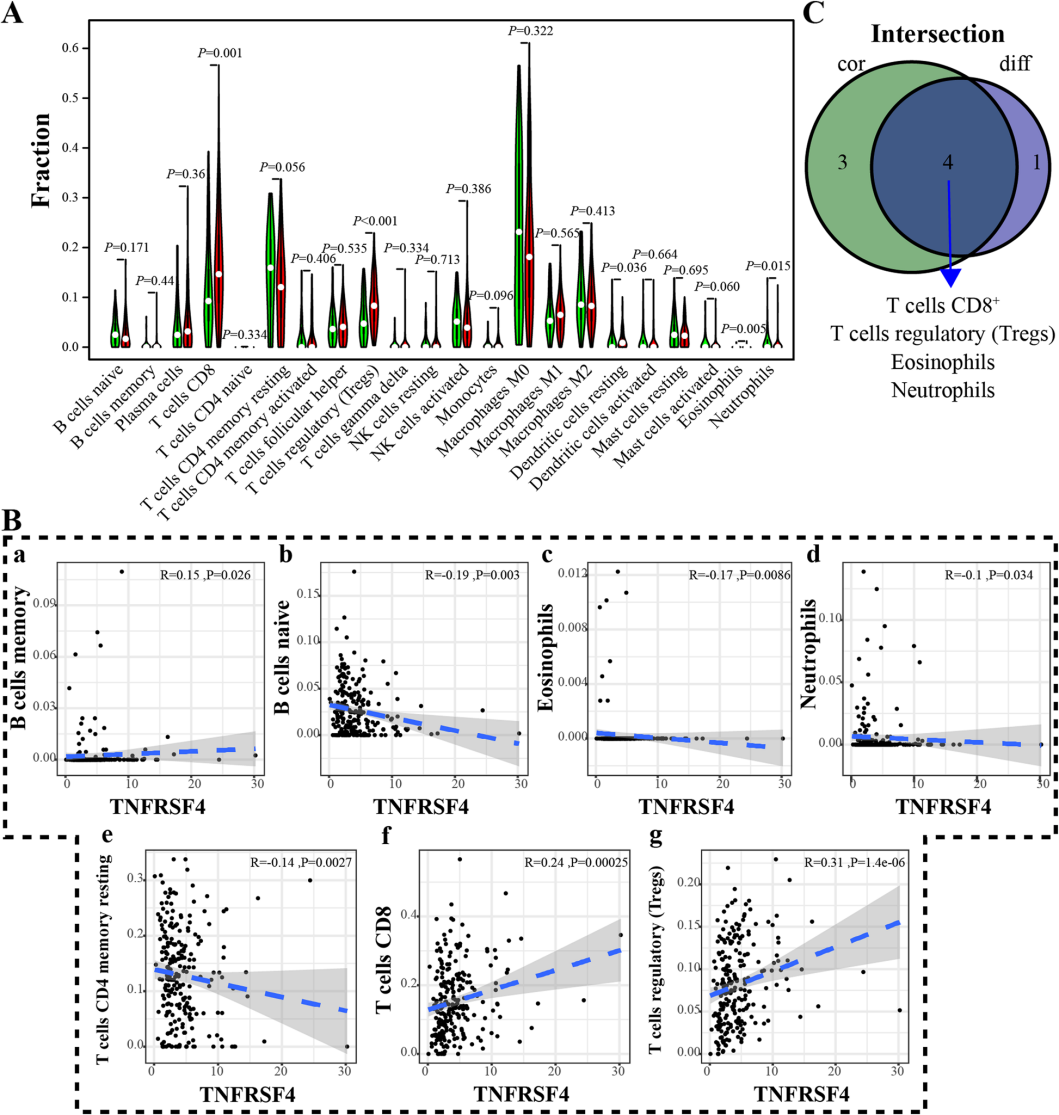
Difference and correlation of TICs proportion with *TNFRSF4* expression. **A** Violin plot showed the percentage differentiation of 22 types of immune cells among EC tumor samples, compared between the high or low-*TNFRSF4* expression groups. **B** Scatter plots showed the correlation of seven kinds of TICs proportion with the *TNFRSF4* expression (*p* < 0.05). The blue line in each plot was a linear fit suggesting the proportion trend of the immune cell as *TNFRSF4* expression altered. **C** Venn plot displayed four kinds of TICs affected by *TNFRSF4* expression, co-determined by difference and correlation analysis as displayed in violin and scatter plots

To verify the primary immune cells affected by *TNFRSF4*, the difference and correlation analysis were carried out, and results demonstrated that a total of 5 kinds of TICs differed between the high and low-*TNFRSF4* expression groups, including CD8^+^ T cells, regulatory T cells, resting dendritic cells, eosinophils, and neutrophils (Fig. 6A). Furthermore, seven kinds of immune cells were correlated with the expression of *TNFRSF4*, as observed in Fig. 6B. In addition, four types of immune cells, including CD8^+^ T cells, regulatory T cells, eosinophils, and neutrophils, were identified to be vitally interconnected with *TNFRSF4* (Fig. 6C). T-cell-mediated immune modulation in the immune microenvironment partly depends on the subsets of T cells. In this part, we analyzed the potential relationship between *TNFRSF4* and the subsets of T cells. Results showed that *TNFRSF4* was positively correlated with subsets of T cells, such as Th1 (R = 0.35), Th17 (R = 0.15) and Treg (R = 0.6), but negative correlated with Th2 (R = − 0.12) (Supplementary Fig. 4D, E). These results hinted that the expression of *TNFRSF4* had much to do with the immune activity of TME in EC.

### Identify the expression of *TNFRSF4* and its correlation with immune-related genes

**Fig. 7:**
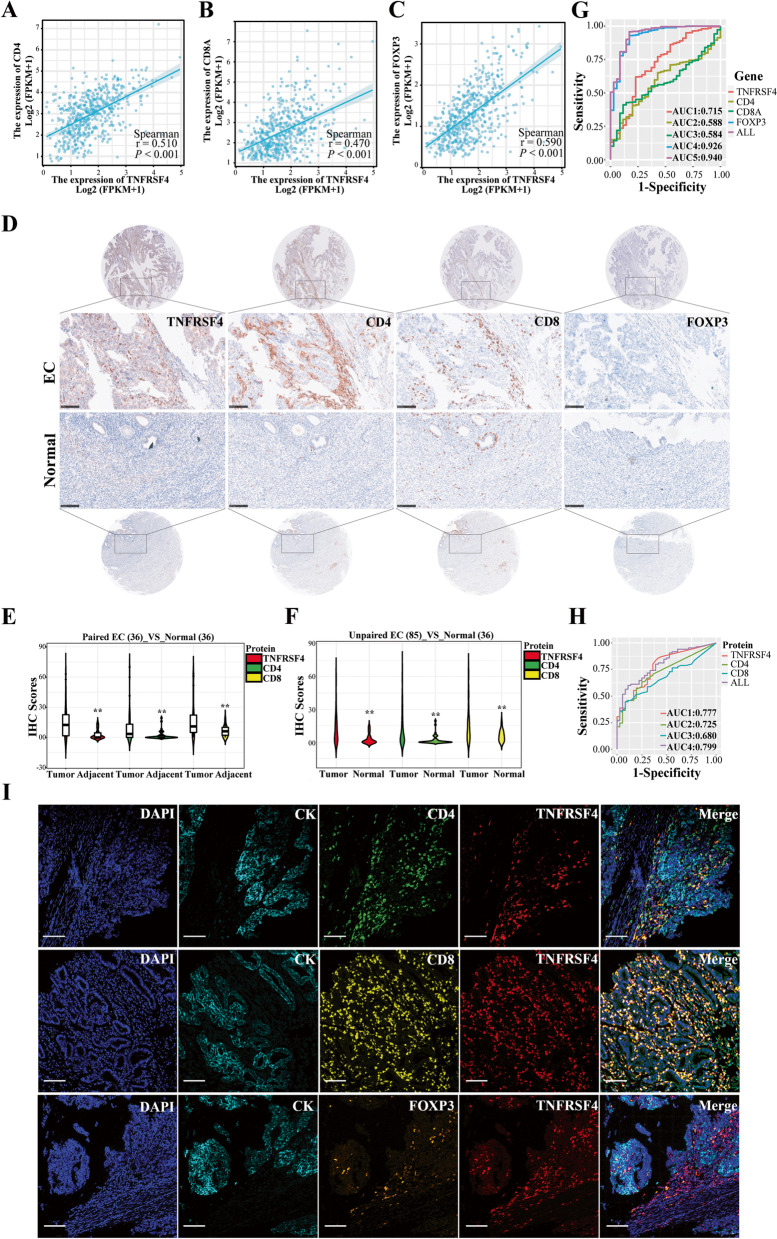
Characterize the co-expression and correlation of *TNFRSF4* with immune-related genes in EC TAMs. **A**, **B**, **C** Correlation of *TNFRSF4* with *CD4*, *CD8A*, and *FOXP3* analyzed from EC-TCGA database. **D** Representative IHC staining for TNFRSF4, CD4, CD8, and FOXP3 in EC and adjacent normal tissue. × 200 magnification (scale bar = 100 μm). **E**, **F** Violin plots visualizing the quantification of TNFRSF4, CD4, and CD8 in EC compared with adjacent normal tissue, 36 cases were included in paired comparison, and 85 ECs and 36 normal tissue were included in unpaired comparison. **G**, **H** ROC curves analyzing the diagnostic performance of *TNFRSF4*, *CD4*, *CD8A*, and *FOXP3* for EC patients in the TCGA and clinical specimens by pROC package. **I** Representative endometrial carcinoma stained with five markers (CK, CD4, CD8, FOXP3, and TNFRSF4). Individual markers were displayed on the left, and merged images were displayed on the right. Scale bar: 100 μm

From the TCGA mRNA expression profiles, we found that the expression of *TNFRSF4* was positively correlated with *CD4* (R = 0.51), *CD8A* (R = 0.47), and *FOXP3* (R = 0.59) (Fig. 7A, B, C). In IHC staining, representative images of TNFRSF4, CD4, CD8, and FOXP3 were exhibited in Fig. 7D**,** TNFRSF4, CD4, and CD8 proteins were over-expressed in EC tissues (mainly on tumor immune infiltrating cells) compared with paired adjacent normal tissues or even unpaired normal tissues (Fig. 7E, F). However, we did not detect any evident expression of FOXP3 at the protein level. Besides, there was a significant statistical correlation of TNFRSF4 with CD4 and CD8, except for clinicopathologic parameters (such as age and histological grade) (Supplementary Table 5). To assess the diagnostic value of *TNFRSF4* in EC, the ROC diagnosis model was performed. Surprisingly, we uncovered that TNFRSF4 had a higher diagnostic significance in either the TCGA dataset (the AUC value = 0.715) (Fig. 7G) or clinical specimens (the AUC value = 0.777) (Fig. 7H), even compared with the diagnostic capability of CD4 or CD8.

Based on the results from IHC, we further carried out m-IHC to comprehensively investigate the relationship among TNFRSF4, CD4, CD8, and FOXP3 in the TME. As shown in Fig. 7I, TNFRSF4, CD4, and CD8 mainly expressed on the tumor stroma and displayed a co-localization pattern observed in both the separate and merged images. Similarly, no reliable fluorescence staining with FOXP3 was observed. Altogether, these data implied the potential role of TNFRSF4 in immune microenvironment remodeling and diagnostic performance for EC.

## Discussion

Given the limited understanding of the details of TME for EC, especially the complicated and volatile characteristics of the immunological microenvironment, in this study, we comprehensively analyzed the alteration of TME and the composition of TICs in EC-based on CIBERSORT algorithms. We then determined *TNFRSF4* and *SIRP4* as immune- and prognosis-related hub genes obtained by the intersection between Cox proportional hazards regression and PPI analysis. Finally, focused on *TNFRSF4*, we found that *TNFRSF4* might be responsible for stably maintaining the immunomodulatory characteristics of TME, thus leading to a better prognosis, which was further validated in the TCGA dataset and clinical sample.

Exploring TME signature genes is a necessary step in uncovering intricate relationships among clinical features and molecular characteristics. As a classical tool, the ESTIMATE algorithm has been used to screen out potential novel biomarkers based on stromal and immune gene sets from each sample in the tumor expression matrix. As known, the higher score estimated in ImmuneScore or StromalScore indicated a higher proportion of corresponding immune or stromal cells in TME. Here, our data demonstrated that ImmuneScore had a more significant correlation with the overall survival rate than StromalScore and ESTIMATEScore, which suggested that TME composition was more critical in modulating cancer progression and prognosis of EC patients. To screen out novel genes related to TME in EC, 587 EC cases from the TCGA database were divided into the high and low-score groups based on the median of the StromalScore and ImmuneScore. Our results showed that 386 DEGs were shared in common by ImmuneScore and StromalScore; it suggested that these DEGs played a vital role in modulating the immunological environment and implied a series of intricate interactions among non-neoplastic cells within the TME that could impact tumor fate [12].

To identify potential biomarkers, the top 40 PPI genes cluster and 16 prognostic-related genes were intersected, leading to *TNFRSF4* was surfaced. *TNFRSF4*, also known as OX40 or CD134, is a co-stimulatory molecule mainly expressed on CD4^+^ and CD8^+^ T cells. Initially, evidence was brought by Paterson, D.J [27], who identified *TNFRSF4* as a specific marker of T-cell activation and survival when cross-linked with its ligand *TNFSF4*. Then emerging evidence highlighted that *TNFRSF4* was a promising therapeutic target for T cell-mediated anti-tumor immunotherapy [28–30]. However, there are still some contradictory sides of *TNFRSF4*, especially since the prognostic effects of TNFRSF4 among different kinds of tumors are often quite different. These paradoxical results could be explained by heterogeneous TME and homeostatic regulation of Tregs [31]. Encouragingly, the latest evidence reinforced the notion that immunotherapy with *TNFRSF4* agonists would trigger a new trend in cancer therapies [32]. For example, *TNFRSF4* stimulation plus a CTLA-4 blockade functioned as a potential therapeutic strategy to eradicate disseminated tumors [33]. In short, *TNFRSF4* might act as a win-win path to reestablish T-cell anti-tumor activity.

Strangely, TNFRSF4, as a second-generation immune checkpoint molecule, has been poorly studied in EC. That encourages us to explore whether *TNFRSF4* can serve as a reliable prognostic biomarker. Our investigation found that transcriptome signatures of immune-related activities were positively correlated with *TNFRSF4* and chiefly enriched in inflammatory signaling pathways, such as IFN-γ response, which is essential for CD8^+^ T lymphocyte-mediated anti-tumor immunity [34]. Immune cell infiltration has held great promise as a new biomarker of prognosis in patients with different types of solid tumors, including endometrial cancer. To further investigate the role of *TNFRSF4* in TME, we firstly estimated the component of TICs in EC samples by the CIBERSORT algorithm. Consistent with previous studies, our results demonstrated that T cells and macrophagocytes were dominant in TME. The proportion of CD8^+^ T cells was positively related to the amount of CD4^+^ memory-activated T cells rather than CD4^+^ memory resting T cells and M0 macrophagocytes. As known, CD4^+^ T cell help is indispensable for sustaining CD8^+^ T cell function during chronic viral infection and engaged in anti-tumor activities by CD8^+^ cytotoxic T cells-dependent apoptosis [35, 36]. Based on our results, further confirming this correlation and evaluating the prognostic significance of *TNFRSF4* in large-scale cohort studies are of great importance.

*TNFRSF4* can orchestrate various subsets of T cells (including CD8^+^ and CD4^+^ T cells, Th1, Th2, Th9, Th17, Tfh, and Tregs [37]) to coordinate the immune microenvironment. For example, the expression of *TNFRSF4* promotes CD8^+^ and CD4^+^ T cell proliferation and survival, enhance the Th1-mediated immune response, argument generation and maintenance of Th2, favor Th9 differentiation and modulate the survival and function of Th17, promote Tfh development, antagonize Treg-mediated immune-suppression through direct inhibition of FOXP3 expression [30, 38, 39]. Treg cells act as immunoregulators characterized by the expression of FOXP3, CD4, and CD25 [40]. Under some conditions, *TNFRSF4* tends to reduce the generation of Tregs by strongly antagonizing TGF-β and IL-10 and inhibiting the antigen mediated-conversion of naive CD4^+^ T cells developed into FOXP3^+^ Tregs [41, 42]. Consistent with that, we find that *TNFRSF4* was negatively correlated with FOXP3 in protein levels from the patient sample but positively related to Treg cells in mRNA levels from the TCGA database. In a word, the effects of *TNFRSF4* on immune modulation depend on the cellular localization and biology function, notably within the immune microenvironment.

Inspired by these results, we further verify the protein expression of TNFRSF4 in clinical specimens by IHC and m-IHC. Our result showed that TNFRSF4 was overexpressed in cancer tissues compared with adjacent normal tissues, and it was mainly located in the TILs within the tumor stroma. It is worth noting that the distinct location of TNFRSF4, as previously described, indicated different prognostic values. For example, the expression of TNFRSF4 on TILs was correlated with favorable prognosis in human cancers, including advanced gastric cancer, non-small cell lung cancer, ovarian carcinoma, malignant melanoma, and colorectal cancer [25, 43–46]. However, the expression on cancer cells was associated with a poorer outcome, including hepatocellular carcinoma and cutaneous squamous cell carcinoma [47, 48]. Furthermore, TNFRSF4-positive cells were also observed to interact with CD4^+^ and CD8^+^ T cells, possibly due to the role of TNFRSF4 in the proliferation of CD4^+^ or CD8^+^ T cells and the survival of antigen-specific memory T cells. Additionally, one unexpected result by ROC curve analysis suggested that TNFRSF4 expression could serve as a potential indicator of favorable diagnosis among patients with EC, and this diagnosis performance was better than both CD4 and CD8.

## Conclusions

In summary, *TNFRSF4* was identified as a novel molecule in EC; its elevated expression tends to signify favorable clinical outcomes and is closely related to the abundance of CD4^+^ and CD8^+^ T cells. As a second immune checkpoint, *TNFRSF4* may have potential implications for the prognosis and immunomodulation of EC patients. Notably, our study suggested that further in-depth understanding the role of *TNFRSF4* might provide new insights into the immunotherapy of EC.

## Supplementary Information

**Additional file 1: Supplementary Figure 1.** The flow diagram of the research design. This flow chart presented a comprehensive bioinformatics analysis and cohort validation to screen out the putative target gene, *TNFRSF4*, and investigate its clinicopathologic significance in EC.

**Additional file 2: Supplementary Figure 2.** Representative images of color schemes for m-IHC. Scale bar: 100 μm.

**Additional file 3: Supplementary Figure 3.** The correlation between Estimate scores with clinicopathologic parameters. A, B, C, D, E, F, G, H, I, J, K M. Distribution of ImmuneScore, StromalScore, and ESTIMATEScore grouped by age, grade, stage and histology, respectively. Comparisons were performed by the Kruskal Wallis rank-sum test.

**Additional file 4: Supplementary Figure 4.** Characterize the expression of S1PR4, and identification of EC immune molecular subtypes. A, B. The expression of *S1PR4* in unpaired and paired ECs and adjacent normal tissue based on TCGA database. C. The distribution of the expression of TNFRSF4 among the four EC molecular subtype. D. Single sample GSEA (ssGSEA) was performed using TCGA-EC gene sets. The enrichment score of ssGSEA was displayed in the heatmap. E. Validating the correlation of TNFRSF4 expression with four kinds of subsets of T cells. The correlation coefficient and *P* values were calculated by Spearman correlation analysis.

**Additional file 5: Supplementary Figure 5.** Independent prognostic marker selected by LASSO Cox regression analysis. A. Lasso coefficient profiles of the 5 co-variates. Each curve was on behalf of a co-variate. B. Partial likelihood deviance was calculated by the cross-validation for the best λ to determine the minimum mean cross-validated error.

**Additional file 6: Supplementary Table 1.** Clinical baseline characteristics of all subjects of the TMAs samples.

**Additional file 7: Supplementary Table 2.** Detailed information of primary antibody for IHC and m-IHC.

**Additional file 8: Supplementary Table 3.** Gene Expression Omnibus datasets involved.

**Additional file 9: Supplementary Table 4.** Results of multivariate analyses for identifying independent survival factors in patients with endometrial cancer.

**Additional file 10: Supplementary Table 5.** Association among TNFRSF4 expression and clinicopathologic parameters, CD4 and CD8 in patients with EC of the validation cohort.

## Data Availability

All data from our study were openly available from the TCGA repository (TCGA-UCEC) and GEO (GSE17025, GSE63678, GSE115810, GSE56087, GSE13003 and GSE146889) database (https://portal.gdc.cancer.gov/; https://www.ncbi.nlm.nih.gov/geo/query/acc.cgi).

## Abbreviations

EC: Endometrial carcinoma
TME: Tumor microenvironment
IHC: Immunohistochemistry
TMAs: Tissue microarrays
TICs: Tumor-infiltrating immune cells
DEGs: Differentially expressed genes
FDR: False discovery rate
FC: Fold change
GO: Gene ontology
KEGG: Kyoto Encyclopedia of Genes and Genomes
PPI: Protein-protein interaction
ssGSEA: Single-sample geneset enrichment analysis
HE: Hematoxylin and eosin
FFPE: Formalin-fixed paraffin-embedded
TILs: Tumor-infiltrating lymphocytes
m-IHC: Multiplex immunohistochemistry
CK: Cytokeratin
BP: Biological process
CC: Cellular component
MF: Molecular function
