# Correction: Identification and validation of TNFRSF4 as a high-profile biomarker for prognosis and immunomodulation in endometrial carcinoma

**DOI:** 10.1186/s12885-022-09692-0

**Published:** 2022-05-30

**Authors:** Heng Ma, Peng-Hui Feng, Shuang-ni Yu, Zhao-Hui Lu, Qi Yu, Jie Chen

**Affiliations:** 1 Department of Pathology, Peking Union Medical College Hospital, Peking Union Medical College and Chinese Academy of Medical Science, Beijing, 100730 China; 2 Department of Obstetrics and Gynecology, Peking Union Medical College Hospital, Peking Union Medical College and Chinese Academy of Medical Science, Beijing, 100730 China

**Correction: BMC Cancer 22, 543 (2022)**

**https://doi.org/10.1186/s12885-022-09654-6**

Following publication of the original article [1], the authors indicated the following error in the equal contribution section. Only Heng Ma and Peng-hui Feng are co-first authors.
