# Supplementary figures and images for: Identification and validation of TNFRSF4 as a high-profile biomarker for prognosis and immunomodulation in endometrial carcinoma
Source: BMC Cancer. 2022 May 13;22:543. doi: 10.1186/s12885-022-09654-6 (PMC9107201; doi:10.1186/s12885-022-09654-6)

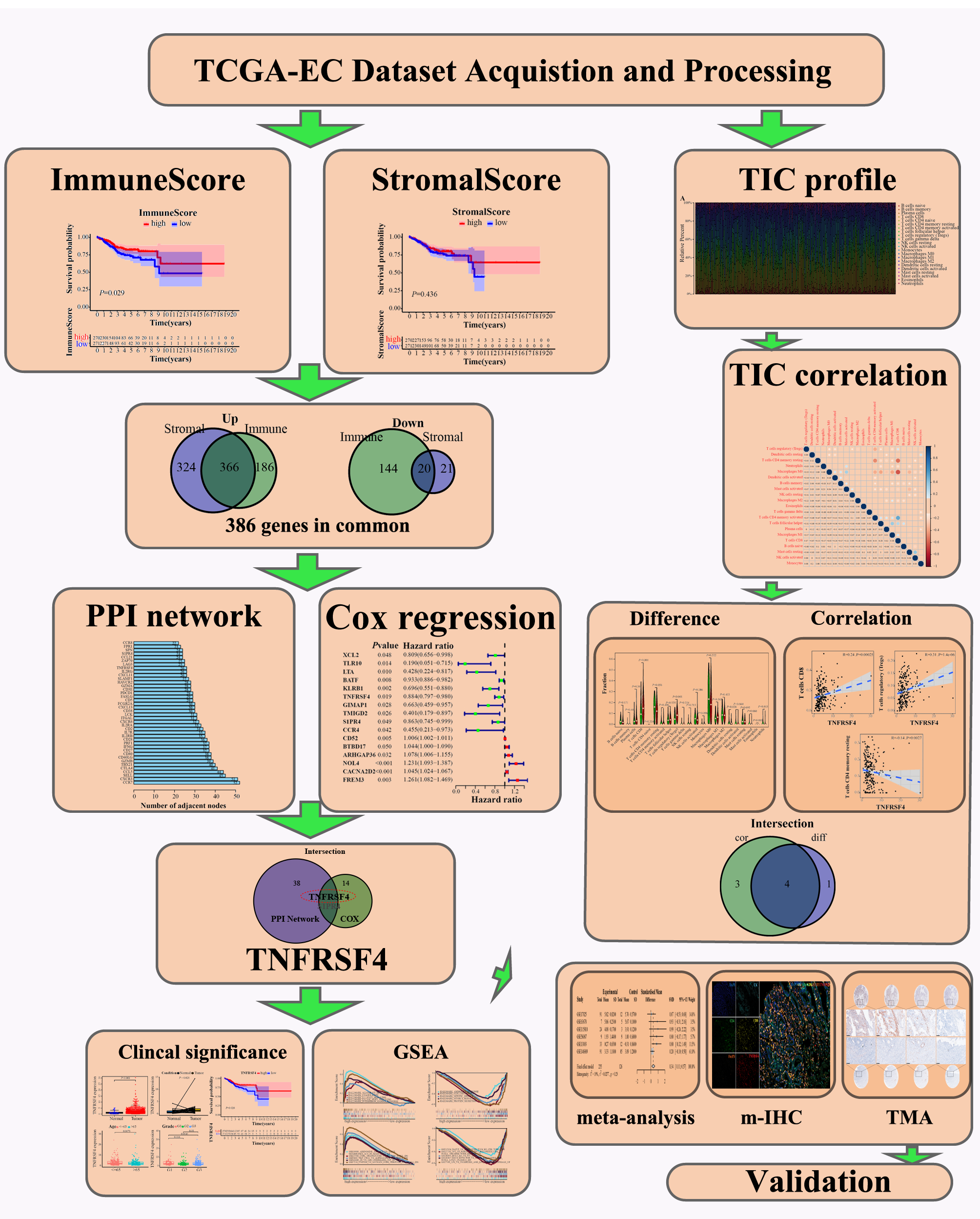

Supplement: Supplementary file 1 — Additional file 1: Supplementary Figure 1. The flow diagram of the research design. This flow chart presented a comprehensive bioinformatics analysis and cohort validation to screen out the putative target gene, TNFRSF4, and investigate its clinicopathologic significance in EC. [file 12885_2022_9654_MOESM1_ESM.tif]

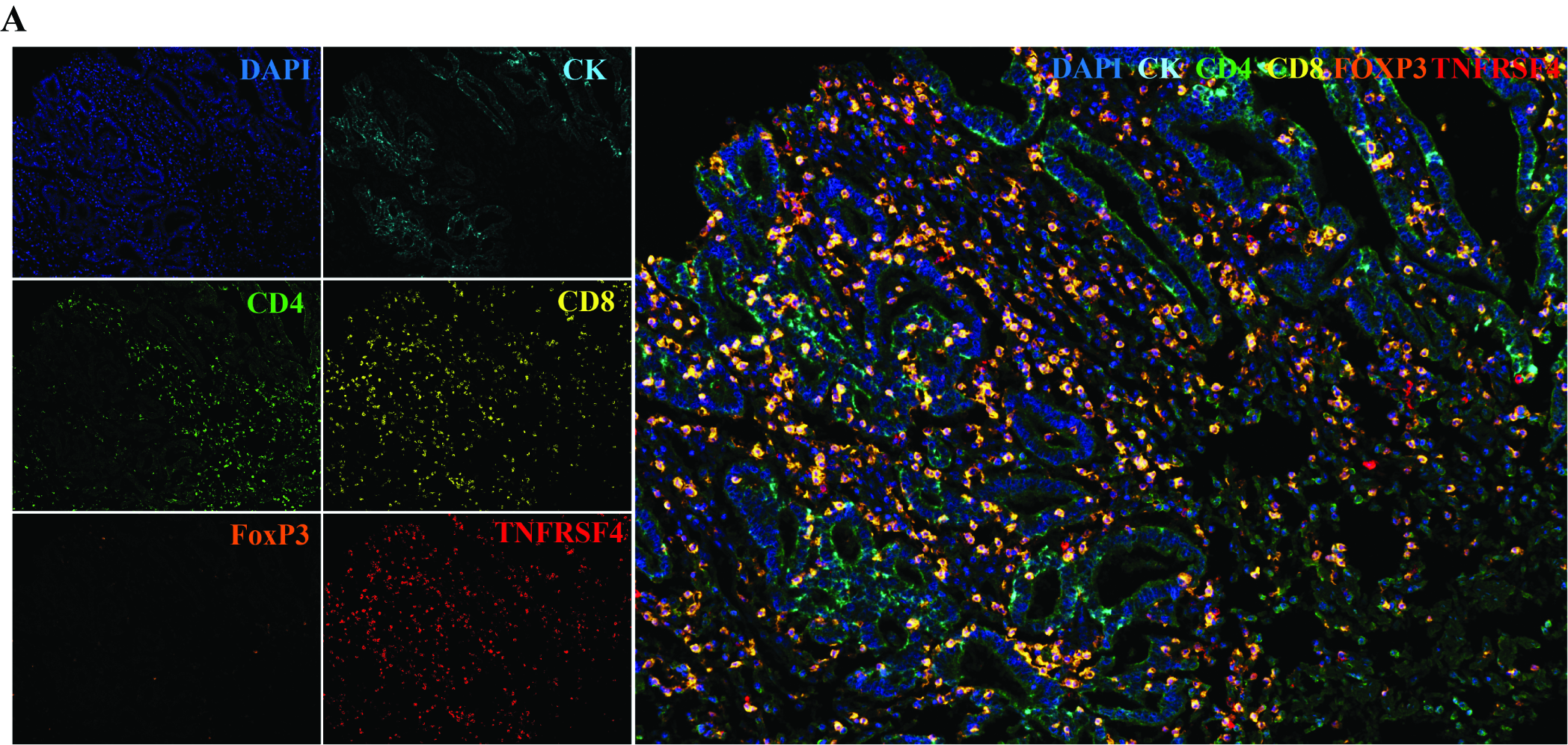

Supplement: Supplementary file 2 — Additional file 2: Supplementary Figure 2. Representative images of color schemes for m-IHC. Scale bar: 100 μm. [file 12885_2022_9654_MOESM2_ESM.tif]

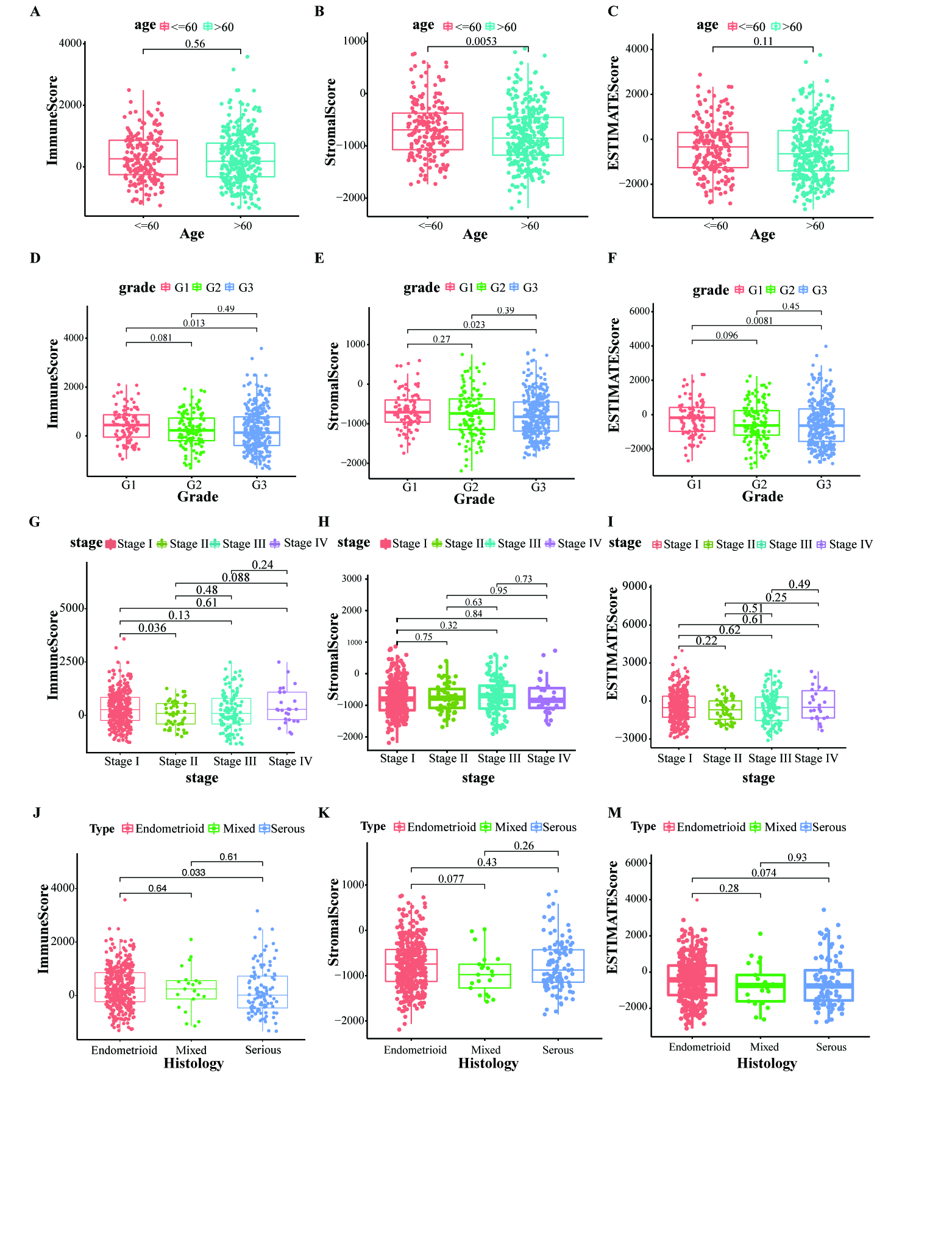

Supplement: Supplementary file 3 — Additional file 3: Supplementary Figure 3. The correlation between Estimate scores with clinicopathologic parameters. A, B, C, D, E, F, G, H, I, J, K M. Distribution of ImmuneScore, StromalScore, and ESTIMATEScore grouped by age, grade, stage and histology, respectively. Comparisons were performed by the Kruskal Wallis rank-sum test. [file 12885_2022_9654_MOESM3_ESM.tif]

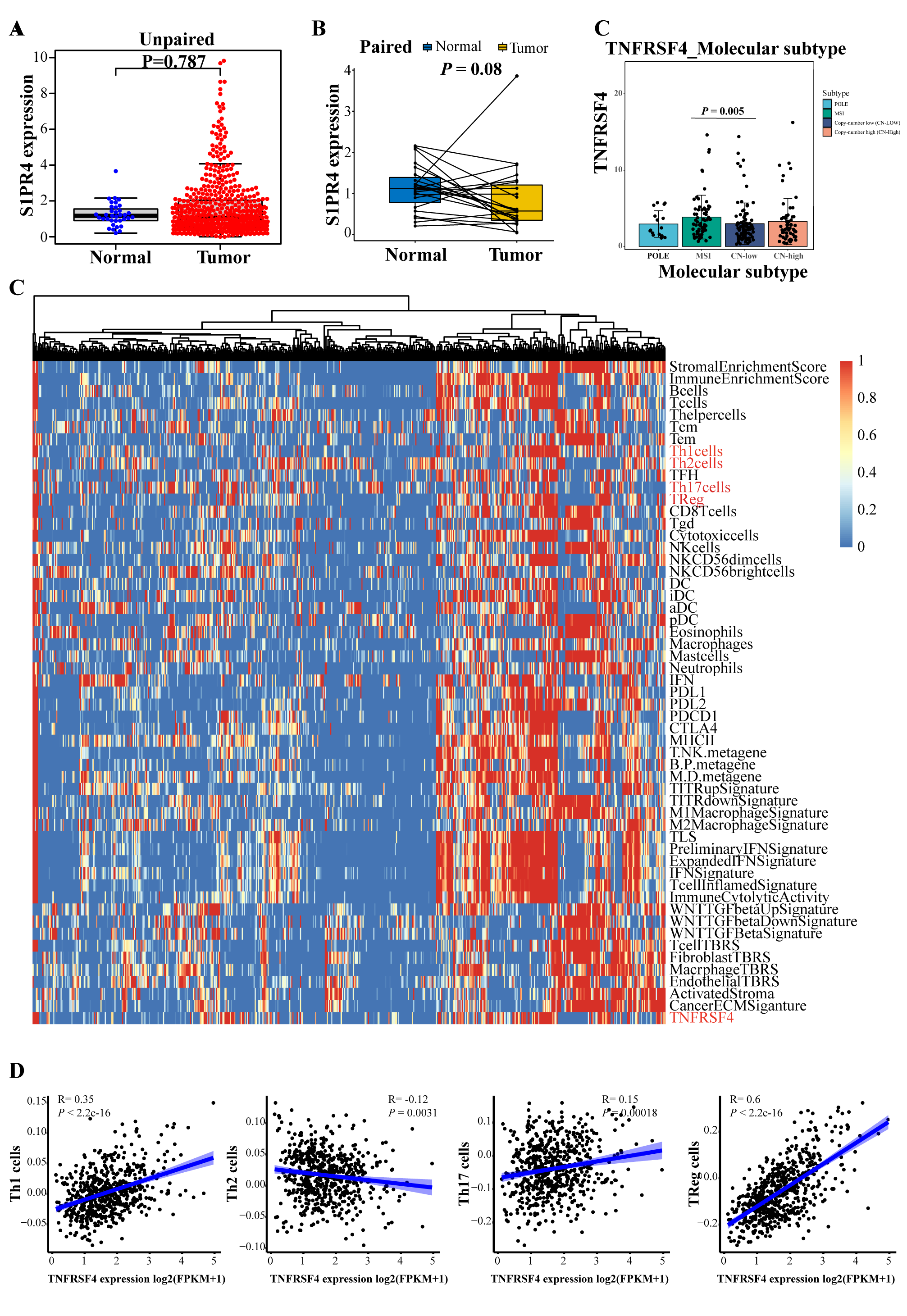

Supplement: Supplementary file 4 — Additional file 4: Supplementary Figure 4. Characterize the expression of S1PR4, and identification of EC immune molecular subtypes. A, B. The expression of S1PR4 in unpaired and paired ECs and adjacent normal tissue based on TCGA database. C. The distribution of the expression of TNFRSF4 among the four EC molecular subtype. D. Single sample GSEA (ssGSEA) was performed using TCGA-EC gene sets. The enrichment score of ssGSEA was displayed in the heatmap. E. Validating the correlation of TNFRSF4 expression with four kinds of subsets of T cells. The correlation coefficient and P values were calculated by Spearman correlation analysis. [file 12885_2022_9654_MOESM4_ESM.tif]

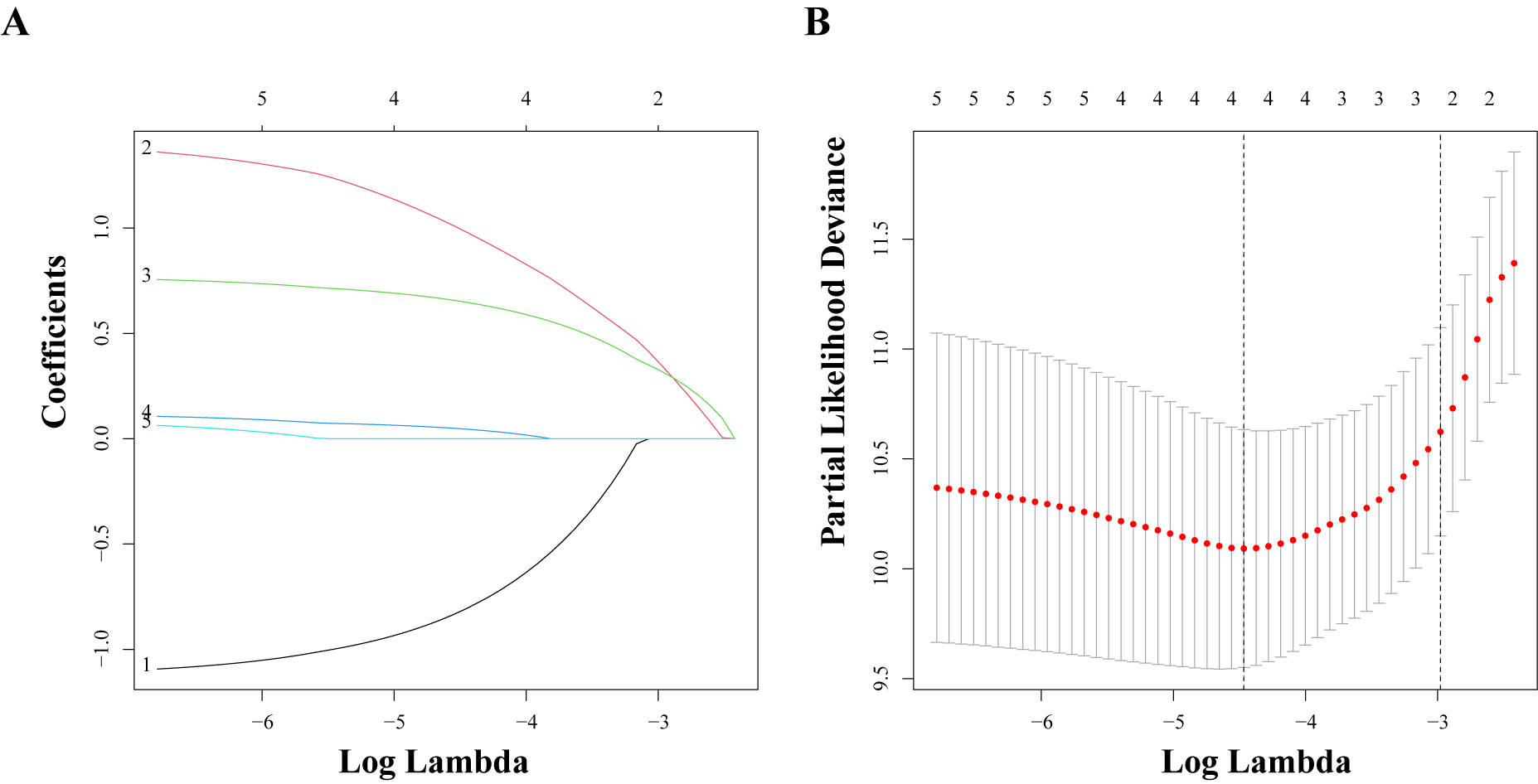

Supplement: Supplementary file 5 — Additional file 5: Supplementary Figure 5. Independent prognostic marker selected by LASSO Cox regression analysis. A. Lasso coefficient profiles of the 5 co-variates. Each curve was on behalf of a co-variate. B. Partial likelihood deviance was calculated by the cross-validation for the best λ to determine the minimum mean cross-validated error. [file 12885_2022_9654_MOESM5_ESM.tif]
