# Supplementary material for: Identification and validation of TNFRSF4 as a high-profile biomarker for prognosis and immunomodulation in endometrial carcinoma
Source: BMC Cancer. 2022 May 13;22:543. doi: 10.1186/s12885-022-09654-6 (PMC9107201; doi:10.1186/s12885-022-09654-6)
Supplement: Supplementary file 6 — Additional file 6: Supplementary Table 1. Clinical baseline characteristics of all subjects of the TMAs samples. [file 12885_2022_9654_MOESM6_ESM.doc]

**Supplementary Table 1. Clinical baseline characteristics of all subjects of the TMAs samples.**

| **Characteristic** | | **Group** | | ***P* value** |
| --- | --- | --- | --- | --- |
|  |  | **EC samples** (n = 85) | **Adjacent normal tissues** (n = 36) |  |
| **Age (years)** | | 57.9 ± 9.5 | 56.7 ± 9.4 | 0.514 |
| **Histological type** | |  |  | - |
|  | Endometrioid adenocarcinoma | 85 (100) | - |  |
| **Pathologic grade** | |  |  | - |
|  | Ⅰ | 12 (14.1) | - |  |
|  | Ⅰ ~ Ⅱ | 18 (21.2) | - |  |
|  | Ⅱ | 32 (37.6) | - |  |
|  | Ⅱ ~ Ⅲ | 12 (14.1) | - |  |
|  | Ⅲ | 11 (12.9) | - |  |
| **Maximum diameter of tumor (cm)** | |  |  | - |
|  | >= 5 | 19 (22.4) | - |  |
|  | 3 ~ 5 | 28 (32.9) | - |  |
|  | <= 3 | 17 (20.0) | - |  |
|  | undefined | 21 (24.7) | - |  |
| **EC:** endometrial carcinoma.  Data were expressed as number (percentage) for categorical variables or mean ± standard deviation for continuous ones.  Student's t test was performed based on normality evaluation for comparisons between groups. | | | | |
