# Supplementary material for: Identification and validation of TNFRSF4 as a high-profile biomarker for prognosis and immunomodulation in endometrial carcinoma
Source: BMC Cancer. 2022 May 13;22:543. doi: 10.1186/s12885-022-09654-6 (PMC9107201; doi:10.1186/s12885-022-09654-6)
Supplement: Supplementary file 7 — Additional file 7: Supplementary Table 2. Detailed information of primary antibody for IHC and m-IHC. [file 12885_2022_9654_MOESM7_ESM.docx]

**Supplementary Table 2.** Detailed information of primary antibody for IHC and m-IHC.

| **Primary Antibody** | **Manufacturer** | **Catalog**  **Number** | **Dilution Ratio**  **for IHC** | **Fluorophore for**  **M-IHC** |
| --- | --- | --- | --- | --- |
| TNRSF4 | Abcam | ab264465 | 1/1000 | Opal 570 |
| CD8A | CST | 85336S | 1/100 | Opal 540 |
| CD4 | Abcam | ab133616 | 1/200 | Opal 520 |
| FOXP3 | CST | 98377S | 1/100 | Opal 650 |
| cytokeratin | CST | 4545S | 1/1000 | Opal 690 |
