# Supplementary material for: Identification and validation of TNFRSF4 as a high-profile biomarker for prognosis and immunomodulation in endometrial carcinoma
Source: BMC Cancer. 2022 May 13;22:543. doi: 10.1186/s12885-022-09654-6 (PMC9107201; doi:10.1186/s12885-022-09654-6)
Supplement: Supplementary file 8 — Additional file 8: Supplementary Table 3. Gene Expression Omnibus datasets involved. [file 12885_2022_9654_MOESM8_ESM.docx]

| **Accession** | **GPL** | **Year** | **Endometrial carcinoma** | | | **Normal control** | | | **Source** |
| --- | --- | --- | --- | --- | --- | --- | --- | --- | --- |
|  |  |  | **n** | **M** | **SD** | **n** | **M** | **SD** |  |
| GSE17025 | GPL570 | 2011 | 91 | 5.82 | 0.82 | 12 | 5.76 | 0.57 | Tissue |
| GSE63678 | GPL571 | 2015 | 7 | 5.86 | 0.23 | 5 | 5.67 | 0.10 | Tissue |
| GSE115810 | GPL96 | 2018 | 24 | 4.08 | 0.17 | 3 | 3.91 | 0.12 | Tissue |
| GSE56087 | GPL11154 | 2015 | 9 | 1.93 | 1.44 | 9 | 1.00 | 0.60 | Tissue |
| GSE13003 | GPL7413 | 2008 | 33 | 0.27 | 0.65 | 12 | -0.31 | 0.86 | Tissue |
| GSE146889 | GPL16791 | 2020 | 91 | 3.33 | 1.18 | 85 | 3.09 | 1.20 | Tissue |
| **M:** mean**, SD:** Standard deviation | | | | | | | | | |

**Supplementary Table 3.** Gene Expression Omnibus datasets involved.
