# Supplementary material for: Identification and validation of TNFRSF4 as a high-profile biomarker for prognosis and immunomodulation in endometrial carcinoma
Source: BMC Cancer. 2022 May 13;22:543. doi: 10.1186/s12885-022-09654-6 (PMC9107201; doi:10.1186/s12885-022-09654-6)
Supplement: Supplementary file 9 — Additional file 9: Supplementary Table 4. Results of multivariate analyses for identifying independent survival factors in patients with endometrial cancer. [file 12885_2022_9654_MOESM9_ESM.docx]

| **Variables** | **Univariate analysis** | |  | **Multivariate analysis** | |
| --- | --- | --- | --- | --- | --- |
|  | **Hazard ratio (95% CI)** | ***P* value** |  | **Hazard ratio (95% CI)** | ***P* value** |
| **TNFRSF4** |  |  |  |  |  |
| low | 1 |  |  |  |  |
| high | 0.541 (0.322-0.912) | **0.021** |  | 0.317 (0.114-0.878) | **0.027** |
| **age** |  |  |  |  |  |
| <=60 | 1 |  |  |  |  |
| >60 | 1.057 (0.613-1.822) | 0.842 |  |  |  |
| **grade** |  |  |  |  |  |
| G3 | 1 |  |  |  |  |
| G2 | 0.264 (0.105-0.662) | **0.005** |  | 0.442 (0.087-2.255) | 0.326 |
| G1 | 0.000 (0.000-Inf) | 0.994 |  | 0.000 (0.000-Inf) | 0.998 |
| **stage** |  |  |  |  |  |
| IV | 1 |  |  |  |  |
| III | 0.375 (0.196-0.720) | **0.003** |  | 0.238 (0.072-0.783) | **0.018** |
| II | 0.109 (0.036-0.331) | **<0.001** |  | 0.068 (0.007-0.613) | **0.017** |
| I | 0.046 (0.021-0.103) | **<0.001** |  | 0.085 (0.024-0.304) | **<0.001** |
| **Histology** |  |  |  |  |  |
| Endometrioid | 1 |  |  |  |  |
| Mixed | 5.197 (2.140-12.623) | **<0.001** |  | 3.701 (0.723-18.947) | 0.116 |
| Serous | 3.504 (1.990-6.168) | **<0.001** |  | 1.402 (0.492-3.991) | 0.527 |
| **Molecular Subtype** |  |  |  |  |  |
| CN_low | 1 |  |  |  |  |
| CN_high | 6.000 (1.710-21.061) | **0.005** |  | 1.715 (0.296-9.943) | 0.547 |
| MSI | 2.137 (0.509-8.965) | 0.299 |  | 2.488 (0.450-13.758) | 0.296 |
| POLE | 0.000 (0.000-Inf) | 0.997 |  | 0.000 (0.000-Inf) | 0.999 |

**Supplementary table 4** Results of multivariate analyses for identifying survival factors in patients with endometrial cancer.
