# Supplementary material for: Identification and validation of TNFRSF4 as a high-profile biomarker for prognosis and immunomodulation in endometrial carcinoma
Source: BMC Cancer. 2022 May 13;22:543. doi: 10.1186/s12885-022-09654-6 (PMC9107201; doi:10.1186/s12885-022-09654-6)
Supplement: Supplementary file 10 — Additional file 10: Supplementary Table 5. Association among TNFRSF4 expression and clinicopathologic parameters, CD4 and CD8 in patients with EC of the validation cohort. [file 12885_2022_9654_MOESM10_ESM.doc]

**Supplementary Table 4.** Association among TNFRSF4 expression and clinicopathologic parameters, CD4 and CD8 in patients with EC of the validation cohort.

| **Parameters** | | **TNFRSF4 expression** | | ***P* value** |
| --- | --- | --- | --- | --- |
|  |  | **High** | **Low** |  |
| **Age (years)** | |  |  | 0.315 |
|  | ≤50 | 11(61.1) | 7(38.9) |  |
|  | >50 | 32(47.8) | 35(55.2) |  |
| **Histological grade** | |  |  | 0.709 |
|  | I | 16(53.3) | 14(46.7) |  |
|  | II-III | 27(49.1) | 28(50.9) |  |
| **CD4** | |  |  | <0.01 |
|  | High | 28(65.1) | 14(34.9) |  |
|  | Low | 15(33.3) | 28(66.7) |  |
| **CD8** | |  |  | <0.001 |
|  | High | 30(73.2) | 11(26.8) |  |
|  | Low | 13(29.5) | 31(70.5) |  |
| Data were expressed as number (Percentage).  Chi-square tests or Fisher's exact test, for categorical variables. | | | | |
